# The Role of Gossiping in Information Dissemination over a Network of Agents

**DOI:** 10.3390/e26010009

**Published:** 2023-12-21

**Authors:** Melih Bastopcu, Seyed Rasoul Etesami, Tamer Başar

**Affiliations:** Coordinated Science Laboratory, University of Illinois Urbana-Champaign, Urbana, IL 61801, USA; bastopcu@illinois.edu (M.B.); etesami1@illinois.edu (S.R.E.)

**Keywords:** information dissemination, gossip networks, gossiping effect, social networks, Markov chains

## Abstract

We consider information dissemination over a network of gossiping agents. In this model, a source keeps the most up-to-date information about a time-varying binary state of the world, and *n* receiver nodes want to follow the information at the source as accurately as possible. When the information at the source changes, the source first sends updates to a subset of m≤n nodes. Then, the nodes share their local information during the *gossiping period*, to disseminate the information further. The nodes then estimate the information at the source, using the majority rule at the end of the gossiping period. To analyze the information dissemination, we introduce a new error metric to find the average percentage of nodes that can accurately obtain the most up-to-date information at the source. We characterize the equations necessary to obtain the steady-state distribution for the average error and then analyze the system behavior under both high and low gossip rates. We develop an adaptive policy that the source can use to determine its current transmission capacity *m* based on its past transmission rates and the accuracy of the information at the nodes. Finally, we implement a clustered gossiping network model, to further improve the information dissemination.

## 1. Introduction

Motivated by many applications—such as autonomous vehicular systems, content advertising on social media, and city emergency-warning systems—information dissemination over the networks has gained significant attention. For instance, in the case of autonomous vehicular systems or city emergency-warning systems, timely critical information, such as accident alerts or tornado warnings, needs to be disseminated as quickly and as accurately as possible. As another example, companies often want to let their potential customers know about their latest products through advertisements over social media. In both of these examples, there is a single information source where the most up-to-date information is disseminated to multiple receivers over time.

In this paper, we consider a communication system with a source and *n* receiver nodes. The source keeps the most recent information about the state of the world, which takes binary values 0 or 1, and changes according to an exponential distribution. Upon each information update, the source wants to let the receiver nodes know about the most recent information. As the source has limited transmission capacity, it cannot send information to more than m≤n nodes, and each information transmission at the source takes an exponentially distributed length of time. After sending updates to *m* nodes, in order to further disseminate information, local information is shared between each pair of receiver nodes, a process we shall refer to as *gossiping.* The gossiping period continues until the information at the source is updated again. At the end of each gossiping period, each receiver node that did not get the most recent information directly from the source comes up with an estimate based on the majority of the information it received from the other nodes. In order to measure the accuracy of the information dissemination at the end of each update cycle, we consider an error metric that takes value 1 for a receiver node that has a different estimate compared to the information at the source.

### 1.1. Related Work

In the gossip-network literature, a model where only one node tries to spread its information to the entire network was considered in [1] and named *single-piece dissemination.* Multi-piece spreading, where all nodes try to spread their individual information to the remaining nodes, was studied in [2]. Moreover, the problem of finding the average of all nodes’ initial information in a gossip network was studied under the framework of *distributed averaging* in [3,4]. The main goal of these works was to analytically characterize either the information spreading time [1,2] or the averaging time [3,4] in the entire network. In all these settings, the information was considered to be *static*, i.e., it did not change over time.

Reference [5] considered the problem of gossiping *dynamic information.* As the gossip network may consist of asynchronous agents where there is no central clock, in order to maintain the information flow in the gossip network, *timestamping* is a commonly used technique, where the agents keep the generation time of their local status updates [6]. During the gossiping phase, information updates among the agents are determined based on whoever has the largest timestamp of particular information, which indicates the *information freshness* of the local agents.

In another line of research, to measure information freshness, the age of information was defined as the difference between the current time and the timestamp of the last status update received by the agents. For a more detailed review of the age of information, we refer to [7,8]. Recently, scaling of the age of information was considered in gossip networks [9,10,11]. In [9], the stochastic hybrid system (SHS) method was used to characterize the version age in arbitrarily connected gossip networks, and scaling of the version age was studied in the symmetric ring and fully connected networks. By using the idea of clustering, scaling of the age of information was further improved in [10]. Then, scaling of the binary freshness metric [12,13,14], which takes either the value 1 when the information is fresh, or 0 otherwise, was studied in the gossip networks in [11].

In all these aforementioned works, the timestamp of the information plays a critical role in determining information dissemination in gossip networks. As the timestamp of the information increases, as new versions of the information are generated, either the size grows without bound—in which case, the agents spend most of their capacity in exchanging large numbers and comparing the values of these large timestamps [5], to determine the freshest information—or, in the case of a bounded timestamp, when overflow happens, the order for information freshness can be lost [6]. In certain applications, an external adversary may interrupt the information flow and alter the timestamp of the information, such that the older versions of the information may be re-branded as fresh information [15]. Recently, the effect of timestomping on age scaling was explored in [16].

Unlike the earlier works on gossip networks, as in [1,2], we considered in this paper a time-varying information source and, instead of tracking the information-spreading time, we studied the average percentage of the nodes that had access to the most recent information at the source before it was updated. Compared to the dynamic information dissemination in [6], in our work we did not use the timestamps of the information. Instead, to maintain the information flow, we used instantaneous signaling from the source to the nodes, to synchronize the nodes. We implemented an information updating mechanism consisting of two phases: in the first phase, only the source could send updates to *m* nodes; in the second phase, i.e., in the gossiping phase, only the nodes could share their local information. Thus, in the gossiping phase, incorrect information in the network could also spread. Works [9,10,11] considered the age of information in gossip networks, where each information update at the source was treated as a new update. In our work, on the other hand, we considered a binary dynamic information source. Thus, the content of the information affected our error metric. Furthermore, in [9,10,11], the nodes updated their information only if they received fresher information. By contrast, in our work, the nodes that did not receive any update directly from the source made decisions based on the majority of the updates that they received from the other nodes. As a result, the error metric and the information updating model that we considered differed from the earlier works in [9,10,11].

The binary information structure appears in real-world applications such as robotic networks, where a group of robots working on a horizontal line wants to decide whether a neighbor robot is in front of the group or behind it. Here, the binary information structure is sufficient to represent the relative position of the robots in the network. Inspired by this example, by using only the sign of the relative states, reference [17] considered a decentralized-online-convex-optimization problem with time-varying loss functions, and reference [18] solved a distributed discrete-time optimization over multi-agent networks. As another notable example, reference [19] considered a model where the actual opinion of the public evolved as a continuous variable in [0,1] but the expressed opinions took only discrete binary values {0,1} that resembled the opinion polls. Motivated by all these examples, in our work, we focused our attention on binary information dissemination as an initial step in analyzing the role of gossiping in information dissemination.

Finally, dissemination of misinformation on social networks has attracted significant interest in recent years. The *network immunization* problem has been considered, to prevent the diffusion of harmful information that can *infect* the network [20,21,22]. More specifically, reference [20] proposed an algorithm that utilizes the community structure for network immunization. Reference [21] proposed a comprehensive solution for the immediate detection and containment of harmful content, aiming to curb its propagation across the network. Reference [22] applied deep neural networks to develop context-aware algorithms that can detect fake news.

### 1.2. Contributions

In this work, we first characterize the equations necessary to obtain the steady-state distribution of the average error (which was also appeared in our preliminary work in [23]). Then, we provide analytical results for the high and low gossip rates. When the gossip rate is high, we show that the probability of obtaining correct information converges to a step function where if the majority of the nodes have the correct information then all the nodes are able to estimate the information correctly with probability 1. In other words, as the gossip rate increases, the information at all nodes becomes mutually available to them, and all the nodes in the network behave like a single node. However, when the gossip rate is low, the gossiping phase can be approximated by either not receiving any updates, in which case the nodes hold on to their prior information, or receiving a single update. Based on this approximation, we characterize analytically the gain obtained through gossiping, and we find an adaptive selection policy for the source, which suggests that the source should send updates to more nodes when the nodes have mostly incorrect information. Then, to further reduce the average error, we implement the idea of clustering, where, instead of sending information to all nodes, the source sends its information only to a smaller number of cluster heads. Then, the cluster heads forward the information to nodes within their clusters. For this network model, we characterize the equations to find the long-term average error at the cluster heads and at the nodes in the clusters. Finally, we provide extensive simulations, to illustrate the effect of gossiping and clustering on information dissemination.

## 2. System Model and Problem Formulation

**Figure 1:**
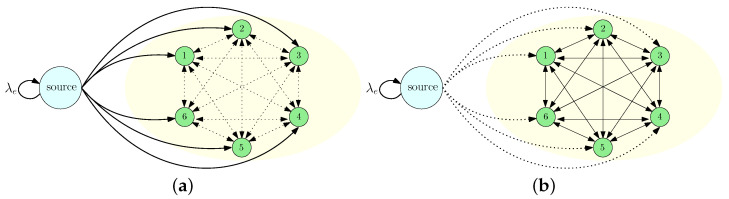
A communication system that consists of a source and fully connected *n* nodes where (**a**) only the source sends updates to the nodes, and (**b**) the nodes share their local information, called the gossiping phase.

We considered an information updating system consisting of a source and *n* receiver nodes, as shown in Figure 1. The source kept the most up-to-date information about a state of the world that took binary values of 0 or 1. The information at the source was updated following a Poisson process with rate $\lambda_{e}$. We defined the time interval between the *j*th and j+1th information update at the source as the *j*th update cycle and denoted it by $I_{j}$. We assumed that the source was able to send instantaneous signals to the nodes. After receiving these signals, the nodes knew that information at the source had been updated, but they did not know what information had been realized at the source. Such instantaneous signalings exist in many practical systems. For example, consider a news provider making news either to support or oppose a topic of interest. After the news is published, the news provider can send an instantaneous notification to its subscribers about the occurrence of the news through push notifications on smart devices or headlines in TV broadcasts or their websites. However, after receiving these notifications, individuals still do not know the actual update until they enter the news provider’s website or watch the TV broadcast. As another application, consider a city emergency-warning system, or anomaly detection in security applications where warning signals can occur over time. As warning signals can also happen due to false alarms, upon receiving such warning signals, individuals do not know whether there is an actual anomaly or not, until further test results can confirm the actual status. Thus, motivated by the aforementioned examples, we utilized the synchronization signal, which can indicate the information update at the source but does not provide any information about the source’s state realization.

We denoted the information at the source at update cycle *j* as $x_{s}\left(j\right)$. For a given $x_{s}\left(j\right)$, the information at the source at the j+1th update cycle was equal to $x_{s}\left(j+1\right)=x_{s}\left(j\right)$ with probability 1−p and to $x_{s}\left(j+1\right)=1-x_{s}\left(j\right)$ with probability *p*, i.e.,

(1) $$\begin{array}{c} P\left(x_{s}\left(j\;+\;1\right)|x_{s}\left(j\right)\right)=\left\{\begin{array}{cc} 1-p, & \mathrm{if}\;x_{s}\left(j+1\right)=x_{s}\left(j\right)\;,\\ p, & \mathrm{if}\;x_{s}\left(j+1\right)=1-x_{s}\left(j\right), \end{array}\right. \end{array}$$

for all *j*, where $0<p<\frac{1}{2}$. As $0<p<\frac{1}{2}$, the nodes kept their state estimation unchanged whenever a new update cycle started. (Our results are extendable to the setting where 0.5<p<1. In this case, the optimal decision taken by each node should be to revert their belief at the beginning of each update cycle.)

The source updated each receiver node according to a Poisson process with rate $\frac{\lambda_{s}}{n}$. In this system, in addition to the update arrivals from the source, each node can share its local information with the other nodes, a process called *gossiping.* Specifically, in this work, we considered a fully connected network where each node was connected to every other node with equal update rates. The total update rate of a node was λ. Thus, in this network, each node updated its neighbor nodes following a Poisson process with rate $\frac{\lambda }{n-1}$. We denoted the information at node *i* at update cycle $I_{j}$ as $x_{i}\left(j\right)$. The nodes wanted to follow the most up-to-date information prevailing at the source as accurately as possible, based on the updates received from the source as well as from the neighbor nodes during an update cycle.

In this paper, we considered an information updating mechanism where at the beginning of each update cycle $I_{j}$ the source sent its current information to *m* nodes where 1≤m≤n, as shown in Figure 1a. Here, we assumed that the source knew (or was able to sense/monitor) the information prevailing at the nodes and, thus, it sent updates to the nodes that carried different information compared to the source. (This approach was motivated especially by online advertisements, whereby companies such as Amazon and Google are able to monitor whether a potential customer is interested in their target products by the customer’s search, view, and click history and, thus, present their advertisements accordingly. They can sense the final opinion of their potential customer by observing the potential customer’s behavior, such as buying an advertised product). During this phase, if the information at the source was updated, then another update cycle started and, thus, the *j*th update cycle could be terminated before sending updates to *m* nodes. If the source sent updates to *m* nodes, it sent another instantaneous signal to start the gossiping among the nodes. Then, we entered the gossiping phase in the update cycle $I_{j}$. During the gossiping phase, illustrated in Figure 1b, the nodes shared their local information with one another. When the information at the source was updated, the gossiping phase ended. At the end of the gossiping period, the nodes that did not get an update directly from the source updated their information based on the majority of the updates they received during the gossiping period. If a node did not get any updates from the source or the other nodes, it kept its local information unchanged. We denoted the information at node *i* at the end of the gossiping period by $x_{i}^{\prime }\left(j\right)$. In order to measure the performance of the information dissemination process, we defined the error metric for node *i* at the update cycle *j* as

(2) $$\begin{array}{c} \Delta_{i}\left(j\right)=\left|x_{s}\left(j\right)-x_{i}^{\prime }\left(j\right)\right|. \end{array}$$

Then, the average estimation error over all the nodes equaled $\Delta \left(j\right)=\frac{1}{n}\sum_{i=1}^{n}\Delta_{i}\left(j\right)$, and the long-term average estimation error over all the nodes was given by

(3) $$\begin{array}{c} \Delta =\lim_{J\to \infty }\frac{1}{J}\sum_{j=1}^{J}\Delta \left(j\right). \end{array}$$

In the next section, we provide detailed analyses to characterize the long-term average error Δ.

## 3. The Long-Term Average Error

In this section, we characterize the long-term average error Δ. Let us consider a generic update cycle $I_{j}$ and, for simplicity of presentation, let us drop the index *j* from the variables in the rest of the analysis. At the beginning of the update cycle, we denote the number of nodes that have the same information as the source by N∈{0,⋯,n}. In this phase, either the source sends an update to a node after an exponential time with the rate $\lambda_{s}$ or the information at the source is updated after an exponential time with the rate $\lambda_{e}$. Thus, the source sends an update to a node with probability $\frac{\lambda_{s}}{\lambda_{s}+\lambda_{e}}$ or the information at the source is updated and the next update cycle starts with probability $\frac{\lambda_{e}}{\lambda_{s}+\lambda_{e}}$. Therefore, during a typical update cycle *I* with N<n−m, the source sends $K_{s}$ updates with the following probability mass function (pmf):

(4) $$\begin{array}{c} P\left(K_{s}=k_{s}|N<n-m\right)=\left\{\begin{array}{cc} {\left(\frac{\lambda_{s}}{\lambda_{s}+\lambda_{e}}\right)}^{k_{s}}\frac{\lambda_{e}}{\lambda_{s}+\lambda_{e}}, & \mathrm{if}\;k_{s}=0,\cdots ,m-1,\\ {\left(\frac{\lambda_{s}}{\lambda_{s}+\lambda_{e}}\right)}^{m}, & \mathrm{if}\;k_{s}\;=\;m. \end{array}\right. \end{array}$$

Similarly, if N≥n−m, we have

(5) $$\begin{array}{c} P\left(K_{s}=k_{s}|N\geq n-m\right)=\left\{\begin{array}{cc} {\left(\frac{\lambda_{s}}{\lambda_{s}+\lambda_{e}}\right)}^{k_{s}}\frac{\lambda_{e}}{\lambda_{s}+\lambda_{e}}, & \mathrm{if}\;k_{s}=0,\cdots ,n-N-1,\\ {\left(\frac{\lambda_{s}}{\lambda_{s}+\lambda_{e}}\right)}^{n-N}, & \mathrm{if}\;k_{s}=n-N. \end{array}\right. \end{array}$$

For an update cycle with N<n−m, the network enters the gossiping phase with probability $P\left(K_{s}=m|N<n-m\right)={\left(\frac{\lambda_{s}}{\lambda_{s}+\lambda_{e}}\right)}^{m}$, which decreases with *m*. In other words, choosing a large *m* decreases the probability of entering the gossiping phase. On the other hand, choosing a small *m* results in sending updates to a small number of nodes and, thus, in the gossiping phase, incorrect information can be spread. Therefore, there is an optimal *m* that achieves the smallest average error Δ.

If the source sends updates to *m* nodes before the information at the source is updated, then the gossiping phase starts. During the gossiping phase, either each node receives an update from the other nodes after an exponential time with rate λ or the information at the source is updated after an exponential time with rate $\lambda_{e}$. As in [24], in the gossiping phase, node *i* receives $K_{i}$ updates with the following pmf:

(6) $$\begin{array}{c} P\left(K_{i}=k_{i}\right)={\left(\frac{\lambda }{\lambda +\lambda_{e}}\right)}^{k_{i}}\frac{\lambda_{e}}{\lambda +\lambda_{e}},\;\;k_{i}=0,1,\cdots . \end{array}$$

In other words, $K_{i}$ has geometric distribution with parameter $\frac{\lambda_{e}}{\lambda +\lambda_{e}}$, i.e., $K_{i}∼\mathrm{Geo}\left(\frac{\lambda_{e}}{\lambda +\lambda_{e}}\right)$.

At the beginning of the gossiping phase, there are N+m nodes with the same information as the source and n−N−m nodes with incorrect information. For the nodes with $x_{i}=x_{s}$, conditioned on the total number of updates $K_{i}=k_{i}$ that they received during the gossiping phase, the distribution of the number of updates that are equal to $x_{s}$ is given by

(7) P(Ri=r|Ki=ki,xi=xs)=kirN+m−1n−1rn−N−mn−1ki−r,r=0,⋯,ki,

where $R_{i}$ is a random variable denoting the number of updates that are equal to $x_{s}$. In other words, for a node *i* that has $x_{i}=x_{s}$, conditioned on $K_{i}=k_{i}$, the random variable $R_{i}$ has a binomial distribution with parameters $\left(k_{i},\frac{N+m-1}{n-1}\right)$, i.e., $R_{i}∼\mathrm{Bin}\left(k_{i},\frac{N+m-1}{n-1}\right)$. Similarly, for the nodes *i* with $x_{i}\neq x_{s}$, we have

(8) P(Ri=r|Ki=ki,xi≠xs)=kirN+mn−1rn−N−m−1n−1ki−r,r=0,⋯,ki.

At the end of the gossiping period, based on the majority of the updates, the nodes *i* that have $x_{s}$ as their prior information estimate the information at the source as $x_{i}^{\prime }=x_{s}$ with probability $P_{T,1}\left(N\right)$, which is given by

(9) $$\begin{array}{cc} P_{T,1}\left(N\right)= & \sum_{k_{i}=1}^{\infty }P\left(R_{i}\geq \left\lfloor \frac{k_{i}}{2}\right\rfloor +1|K_{i}=k_{i},x_{i}=x_{s}\right)P\left(K_{i}=k_{i}\right)\\ \; & +\frac{1}{2}\sum_{k_{i}=1}^{\infty }P\left(R_{i}=k_{i}|K_{i}=2k_{i},x_{i}=x_{s}\right)P\left(K_{i}=2k_{i}\right)+P\left(K_{i}=0\right). \end{array}$$

We note that the first summation term in (9) corresponds to the case where a node receives a strictly higher number of $x_{s}$ during the gossiping period. The second summation term in (9) refers to the case where a node receives an equal number of $x_{s}$ and $1-x_{s}$. In this case, a node estimates the information as either $x_{s}$ or $1-x_{s}$ with equal probabilities. If a node does not get any updates during the gossiping phase, it keeps its current information that is given by the last term in (9). Similarly, for a node *i* that has prior information $x_{i}\neq x_{s}$, we can derive an expression for the probability of updating its information to $x_{s}$, denoted by $P_{T,2}\left(N\right)$, as

(10) $$\begin{array}{cc} P_{T,2}\left(N\right)= & \sum_{k_{i}=1}^{\infty }P\left(R_{i}\geq \left\lfloor \frac{k_{i}}{2}\right\rfloor +1|K_{i}=k_{i},x_{i}\neq x_{s}\right)P\left(K_{i}=k_{i}\right)\\ & +\frac{1}{2}\sum_{k_{i}=1}^{\infty }P\left(R_{i}=k_{i}|K_{i}=2k_{i},x_{i}\neq x_{s}\right)P\left(K_{i}=2k_{i}\right). \end{array}$$

Note that this expression is identical to that in (9), except that in the summations we use the probabilities $P(R_{i}=r|K_{i}=k_{i},x_{i}\neq x_{s})$ given in (8) and that $P(K_{i}=0)$ is excluded. In the next theorem, we state the long-term average error.

**Theorem 1.**  *Under the proposed gossiping network, the long-term average error* Δ *is given by*

(11) $$\begin{array}{c} \Delta =\sum_{j=0}^{n}\sum_{n^{\prime \prime }=0}^{n}\left(\pi_{0,j}+\pi_{1,j}\right)P\left(N^{\prime \prime }=n^{\prime \prime }|N=j\right)\frac{n-n^{\prime \prime }}{n}, \end{array}$$

*where $P(N^{\prime \prime }=n^{\prime \prime }|N=j)$ is provided in (13) and $\pi =[\pi_{0,0},\cdots ,\pi_{0,n},\pi_{1,0},\cdots ,\pi_{1,n}]$ is the row vector of steady-state probabilities of the Markov chain over the state space $\left(x_{s},N\right)$. The unique stationary distribution is given by the solution of π=πP for a stochastic matrix $P\;\in \;R^{2(n\;+1)\times 2(n\;+1)}\;$, where the transition probabilities of P are given later in (14).*

**Proof.**  We note that at the end of an update cycle with a gossiping phase, *m* nodes that obtain information directly from the source will have $x_{i}^{\prime }=x_{s}$ (In the gossiping phase, these nodes send information to other nodes with rate λ, but they do not update their information based on the updates received from the other nodes). There are *N* nodes that have prior information $x_{s}$. These nodes will update their information to $x_{i}^{\prime }=x_{s}$ with probability $P_{T,1}\left(N\right)$ and to $x_{i}^{\prime }=1-x_{s}$ with probability $1-P_{T,1}\left(N\right)$. Thus, the total number of nodes that update their information to $x_{s}$, denoted by $N_{1}^{\prime }$, has the binomial distribution $N_{1}^{\prime }∼\mathrm{Bin}\left(N,P_{T,1}\left(N\right)\right)$. On the other hand, there are n−N−m nodes that have prior information $1-x_{s}$. At the end of the gossiping phase, these nodes will update their information to $x_{i}^{\prime }=x_{s}$ with probability $P_{T,2}\left(N\right)$ and to $x_{i}^{\prime }=1-x_{s}$ with probability $1-P_{T,2}\left(N\right)$. Thus, the total number of nodes that change their information to $x_{s}$, denoted by $N_{2}^{\prime }$, obeys the binomial distribution $N_{2}^{\prime }∼\mathrm{Bin}\left(n-N-m,P_{T,2}\left(N\right)\right)$. Therefore, at the end of the gossiping period, the total number of the nodes that have $x_{s}$ is equal to $m+N^{\prime }$, where $N^{\prime }=N_{1}^{\prime }+N_{2}^{\prime }$ has the following pmf:

(12) $$\begin{array}{c} P\left(N^{\prime }=n^{\prime }\right)=\sum_{\ell_{1}=\ell_{lower}}^{\ell_{upper}}P\left(N_{1}^{\prime }=\ell_{1}\right)P\left(N_{2}^{\prime }=n^{\prime }-\ell_{1}\right), \end{array}$$

for $n^{\prime }=0,\cdots ,n-m$, where $\ell_{lower}=\max\left\{0,n^{\prime }+N+m-n\right\}$ and $\ell_{upper}=\min\left\{N,n^{\prime }\right\}$.

Next, let us define $N^{\prime \prime }\left(j\right)$ to be the number of nodes that have the same information with the source at the end of the update cycle $I_{j}$, i.e., $x_{i}^{\prime }\left(j\right)=x_{s}\left(j\right)$. If the update cycle $I_{j}$ ends before entering the gossiping phase, then either $N\left(j\right)<n-m,K_{s}<m$ or N(j)≥n−m. In these cases, the source sends updates to $k_{s}$ nodes with probability distributions given in (4) and (5), respectively. If the source is able to send updates to *m* nodes, then the gossiping phase starts and, as a result, $N^{\prime \prime }\left(j\right)=m+n^{\prime }$ nodes will have $x_{s}\left(j\right)$ with probabilities $P\left(K_{i}=m\right)P\left(N^{\prime }=n^{\prime }\right)$, where $n^{\prime }=0,\cdots ,n-m$. Thus, the probability distribution of $N^{\prime \prime }$ for a given *N* is given by

(13) $$\begin{array}{c} \;\;\;\;\;\;P\left(N^{\prime \prime }\;=\;n^{\prime \prime }|N\right)\;=\;\left\{\begin{array}{cc} P(K_{s}=k_{s}|N<n-m), & \mathrm{if}\;n^{\prime \prime }=k_{s}+N<m,\;\\ P\left(K_{s}=m|N<n\;-\;m\right)P\left(N^{\prime }=n^{\prime }\right), & \mathrm{if}\;m\leq n^{\prime \prime }\;=m\;+\;n^{\prime }<N,\\ P(K_{s}\;=\;n^{\prime \prime }\;-\;N|N<n\;-\;m)\\ +P\left(K_{s}\;=\;m|N<n\;-\;m\right)P\left(N^{\prime }=n^{\prime \prime }\;-\;m\right), & \mathrm{if}\;m\leq N\leq n^{\prime \prime }<N\;+\;m,\\ P\left(K_{s}=m|N<n-m\right)P\left(N^{\prime }=n^{\prime \prime }-m\right), & \mathrm{if}\;N+m\leq n^{\prime \prime }\leq n,\\ P(K_{s}=n^{\prime \prime }-N|N\geq n-m), & \mathrm{if}\;n-m\leq N\leq n^{\prime \prime }\leq n. \end{array}\right. \end{array}$$

With the pmf of $N^{\prime \prime }$ as provided in (13), we can fully characterize the transition probabilities of going from *N* nodes that have $x_{s}$ at the beginning of an update cycle to $N^{\prime \prime }$ nodes that have $x_{s}$ at the end of that update cycle. Now let us consider a Markov chain over the state space $\left(x_{s},N\right)$, where by abuse of notation we label the first n+1 states (0,0),(0,1),…,(0,n) by 1,2,…,n+1, and the last n+1 states (1,0),(1,1),…,(1,n) by n+2,n+3,…,2n+2. We can then represent the transition probabilities between different states a,b∈{1,2,…,2n+2}, using a stochastic matrix P, where $P_{a,b}$ denotes the probability of moving from state *a* to state *b* and is given by

(14) $$\begin{array}{c} \;\;\;P_{a,b}\;\;=\;\;\left\{\begin{array}{c} \left(1-p\right)P\left(N^{\prime \prime }=b-1|N=a-1\right),\;\mathrm{if}\;1\leq a\leq n+1,\;1\leq b\leq n+1,\;\\ \frac{p}{1-p}P_{a,2n+3-b},\;\mathrm{if}\;1\leq a\leq n+1,\;n+1\leq b\leq 2(n+1),\;\\ \frac{p}{1-p}P_{a,2n+3-b},\;\mathrm{if}\;n+1\leq a\leq 2(n+1),\;1\leq b\leq n+1,\;\\ \left(1\;-\;p\right)P\left(N^{\prime \prime }\;=\;b\;-\;n\;-\;2|N\;=\;a\;-\;n\;-\;2\right),\;\mathrm{if}\;n\;+\;1\;\leq \;a\;\leq \;2(n\;+\;1),\;n\;+\;1\;\leq \;b\;\leq \;2(n\;+\;1). \end{array}\right.\; \end{array}$$

We note that the stochastic matrix P in (14) is irreducible, as every state *b* is accessible from any state *a* in a finite update cycle duration. As $P_{a,a}>0$ for all *a* in (14), the Markov chain induced by P is also aperiodic. Thus, the above Markov chain admits a unique stationary distribution given by the solution of π=πP, such that $\sum_{i=0}^{1}\sum_{j=0}^{n}\pi_{ij}=1$, $\pi_{ij}\geq 0,\forall i,j$. Finally, we characterize the long-term average error among all the nodes by (11). □

In the following section, we proceed to approximate the probabilities $P_{T,1}\left(N\right)$ and $P_{T,2}\left(N\right)$ provided in this section, to understand the effect of gossiping better when the gossip rate λ is low and high compared to the information change rate at the source $\lambda_{e}$.

## 4. Analysis for High and Low Gossip Rates

In this section, we develop approximations for $P_{T,1}\left(N\right)$ and $P_{T,2}\left(N\right)$, which are the probabilities of choosing $x_{s}$ at the end of a gossiping period when the nodes have the same prior information with the source and when they do not, respectively. First, by assuming sufficiently large *n* and *N*, we can approximate the conditional pmfs for $R_{i}$ given in (7) and (8) by the binomial distribution $P\left(R_{i}|K_{i}=k_{i}\right)∼\mathrm{Bin}\left(k_{i},\frac{N+m}{n}\right)$. Let us denote the corresponding $P_{T,1}\left(N\right)$ and $P_{T,2}\left(N\right)$ obtained by substituting this binomial approximation into (9) and (10) by ${\hat{P}}_{T,1}\left(N\right)$ and ${\hat{P}}_{T,2}\left(N\right)$, respectively. As ${\hat{P}}_{T,1}\left(N\right)={\hat{P}}_{T,2}\left(N\right)+P\left(K_{i}=0\right)$, for the rest of this section we will only approximate ${\hat{P}}_{T,2}\left(N\right)$, and can find the probability ${\hat{P}}_{T,1}\left(N\right)$ accordingly. Next, for sufficiently large values of $k_{i}$, we can approximate $P(R_{i}\geq \frac{k_{i}}{2}|K_{i}=k_{i})$ as

(15) $$\begin{array}{c} P\left(R_{i}\geq \frac{k_{i}}{2}|K_{i}=k_{i}\right)\approx Q\left(\sqrt{k_{i}}A\left(N\right)\right), \end{array}$$

where $A\left(N\right)\;=\;\frac{\frac{1}{2}-\frac{N+m}{n}}{\sqrt{\frac{N+m}{n}\left(1\;-\;\frac{N+m}{n}\right)}}$ and $Q\left(x\right)\;=\;\frac{1}{\sqrt{2\pi }}\int_{x}^{\infty }e^{-\frac{u^{2}}{2}}\;du$. We note that (15) is due to the normal approximation of binomial distribution by using the central limit theorem (CLT). In the following proposition, we show that ${\hat{P}}_{T,2}\left(N\right)$ can be approximated by a summation of *Q*-functions.

**Proposition 1.**  *When λ is sufficiently large compared to $\lambda_{e}$, ${\hat{P}}_{T,2}\left(N\right)$ can be approximated by*

(16) $$\begin{array}{c} P_{T,app}\left(N\right)=\sum_{k_{i}=1}^{\infty }Q\left(\sqrt{k_{i}}A\left(N\right)\right)P\left(K_{i}=k_{i}\right). \end{array}$$

**Proof.**  Using the CLT, there exists a sufficiently large *K*, such that the difference between the probabilities $P(R_{i}\geq \frac{k_{i}}{2}|K_{i}=k_{i})$ and $Q\left(\sqrt{k_{i}}A\left(N\right)\right)$ is smaller than ϵ>0. Then, we have

$$\begin{array}{c} |{\hat{P}}_{T,2}\left(N\right)\;-\;P_{T,app}\left(N\right)|\;\leq \;\sum_{k_{i}=1}^{K}|P\left(R_{i}\geq \frac{k_{i}}{2}|K_{i}=k_{i}\right)\;-\;Q\;\left(\sqrt{k_{i}}A\left(\;N\;\right)\;\right)|P\left(K_{i}\;=\;k_{i}\right)\;+\;\epsilon {\left(\frac{\lambda }{\lambda \;+\;\lambda_{e}}\right)}^{K+1}\;\;, \end{array}$$

where $P\left(K_{i}\;=\;k_{i}\right)={\left(\frac{\lambda }{\lambda +\lambda_{e}}\right)}^{k_{i}}\frac{\lambda_{e}}{\lambda +\lambda_{e}}$, for $k_{i}=0,\cdots ,\infty$. The above expression can be further upper-bounded by

$$\begin{array}{c} |{\hat{P}}_{T,2}\left(N\right)\;-\;P_{T,app}\left(N\right)|\leq 1-{\left(\frac{\lambda }{\lambda \;+\;\lambda_{e}}\right)}^{K+1}\;\;\;\;\;\;+\epsilon {\left(\frac{\lambda }{\lambda \;+\;\lambda_{e}}\right)}^{K+1}\;\;. \end{array}$$

As the term $1-{\left(\frac{\lambda }{\lambda \;+\;\lambda_{e}}\right)}^{K+1}$ can be made smaller than ϵ by choosing $\lambda >\frac{\lambda_{e}{\left(1-\epsilon \right)}^{1/(K+1)}}{1-{\left(1-\epsilon \right)}^{1/(K+1)}}$, the difference between ${\hat{P}}_{T,2}\left(N\right)$ and $P_{T,app}\left(N\right)$ can be smaller than 2ϵ for every ϵ>0 by choosing sufficiently large λ. □

Next, we show that ${\hat{P}}_{T,2}\left(N\right)$ can be approximated by the summation of *Q*-functions when λ is sufficiently large. In the following proposition, we show that as λ→∞ the probability ${\hat{P}}_{T,2}\left(N\right)$ converges to a step function.

**Proposition 2.**  *As λ→∞ the probability ${\hat{P}}_{T,2}\left(N\right)$ converges to a step function given by*

(17) $$\begin{array}{c} \lim_{\lambda \to \infty }{\hat{P}}_{T,2}\left(N\right)\approx \left\{\begin{array}{cc} 0, & when\;\frac{N+m}{n}<\frac{1}{2},\\ \frac{1}{2}, & when\;\frac{N+m}{n}=\frac{1}{2},\\ 1, & when\;\frac{N+m}{n}>\frac{1}{2}. \end{array}\right. \end{array}$$

**Proof.**  First, we consider the case when $\frac{N+m}{n}<\frac{1}{2}$. In this case, we note that $Q\left(\sqrt{k_{i}}A\left(N\right)\right)$ is a decreasing function of $k_{i}$. Thus, for any arbitrary $\epsilon_{1}>0$, there exists an *L*, such that $Q\left(\sqrt{k_{i}}A\left(N\right)\right)<\epsilon_{1},\;\forall k_{i}>L$. Therefore, we have

$$\begin{array}{c} {\hat{P}}_{T,2}\left(N\right)<\sum_{k_{i}=1}^{L}Q\left(\sqrt{k_{i}}A\left(N\right)\right)P\left(K_{i}=k_{i}\right)+\epsilon_{1}{\left(\frac{\lambda }{\lambda +\lambda_{e}}\right)}^{L+1}. \end{array}$$

As $Q\left(\sqrt{k_{i}}A\left(N\right)\right)<\frac{1}{2}$ for $k_{i}\geq 1$, as in the proof of Proposition 1, by choosing sufficiently large λ, one can show that ${\hat{P}}_{T,2}\left(N\right)<2\epsilon_{1}$. Thus, if $\frac{N+m}{n}<\frac{1}{2}$, we have $\lim_{\lambda \to \infty }{\hat{P}}_{T,2}\left(N\right)=0$.

Next, we consider the case when $\frac{N+m}{n}>\frac{1}{2}$. As Q(x)=1−Q(−x) for all *x*, we have

$$\begin{array}{c} {\hat{P}}_{T,2}\left(N\right)=\sum_{k_{i}=1}^{\infty }\left(1-Q\left(-\sqrt{k_{i}}A\left(N\right)\right)\right)P\left(K_{i}=k_{i}\right). \end{array}$$

Note that $Q\left(-\sqrt{k_{i}}A\left(N\right)\right)$ is a decreasing function of $k_{i}$. Thus, for any $\epsilon_{2}>0$, there exists a large $\hat{L}$, such that $Q\left(-\sqrt{k_{i}}A\left(N\right)\right)<\epsilon_{2}$. Therefore, we can write

$$\begin{array}{c} {\hat{P}}_{T,2}\left(N\right)>\frac{\lambda }{\lambda +\lambda_{e}}-\sum_{k_{i}=1}^{\hat{L}}Q\left(-\sqrt{k_{i}}A\left(N\right)\right)P\left(K_{i}=k_{i}\right)-\epsilon_{2}{\left(\frac{\lambda }{\lambda +\lambda_{e}}\right)}^{\hat{L}+1}. \end{array}$$

Now, similar to the first part of the proof, we can show that ${\hat{P}}_{T,2}\left(N\right)>\frac{\lambda }{\lambda +\lambda_{e}}-2\epsilon_{2}$ by selecting a sufficiently large λ. Thus, when $\frac{N+m}{n}>\frac{1}{2}$, we have $\lim_{\lambda \to \infty }{\hat{P}}_{T,2}\left(N\right)=1$. Finally, when $\frac{N+m}{n}=\frac{1}{2}$, we note that the A(N) terms in (16) become 0, which implies ${\hat{P}}_{T,2}\left(N\right)\approx P_{T,app}\left(N\right)=\frac{1}{2}$. □

In Proposition 2, we showed that when the gossip rate λ is sufficiently large, the nodes start to have access to information from all other nodes. As a result, all the nodes in the network collectively start to behave like a single node, where at the end of a gossiping period the information is updated based on the majority of the information at all nodes. In other words, if the majority of the nodes have the same information as the source, which happens if $\frac{N+m}{n}>\frac{1}{2}$, all the nodes update their information to $x_{s}$ and, thus, they will have the same information as the source at the end of the gossiping period. On the other hand, when the majority of the nodes have the incorrect information $1-x_{s}$, which happens if $\frac{N+m}{n}<\frac{1}{2}$, then all the nodes will have the incorrect information at the end of the gossiping period. Therefore, when the information at the source changes frequently (i.e., $\lambda_{e}$ is large) and the source has limited total update rate capacity (i.e., $\lambda_{s}$ is small), a high gossip rate λ can cause incorrect information to disseminate in the network. As a result, gossiping can be harmful in these scenarios. On the other hand, when the source has high transmission rates, at each update cycle, it is enough for the source to send its information to the number of nodes that achieves the majority, i.e., $\frac{N+m}{n}>\frac{1}{2}$. After that, the remaining nodes can obtain the correct information during the gossiping phase. Thus, when the source has enough transmission rate, high gossip rates among the nodes can be utilized by sending the updates to at most half the network.

Next, we consider the case in which the gossip rate λ is relatively low compared to the rate of information change at the source, $\lambda_{e}$. When the gossip rate is low, the nodes either do not get any updates, in which case they hold on to their prior information, or they mostly get only one update from the other nodes and, hence, update their information based on the only received update. In the following proposition, we approximate the probability ${\hat{P}}_{T,2}\left(N\right)$ when λ is low.

**Proposition 3.**  *When λ is sufficiently small, the probability ${\hat{P}}_{T,2}\left(N\right)$ can be approximated by*

(18) $$\begin{array}{c} P_{T,app}^{low}\left(N\right)=\frac{\lambda }{\lambda +\lambda_{e}}\frac{N+m}{n}. \end{array}$$

**Proof.**  When λ is sufficiently low, the nodes may not receive any updates or receive a single update packet from the other nodes in the gossiping phase. Thus, the nodes that have the incorrect information $1-x_{s}$ as prior information obtain $x_{s}$ with probability $\left(1-P\left(K_{i}=0\right)\right)\frac{N+m}{n}$, which is equal to

$$\begin{array}{c} P_{T,app}^{low}\left(N\right)=\frac{\lambda }{\lambda +\lambda_{e}}\frac{N+m}{n}. \end{array}$$

Next, we consider the difference between ${\hat{P}}_{T,2}\left(N\right)$ and $P_{T,app}^{low}\left(N\right)$, which is given by

$$\begin{array}{c} |{\hat{P}}_{T,2}\left(N\right)-P_{T,app}^{low}\left(N\right)|\leq \sum_{k_{i}=2}^{\infty }P\left(R_{i}\geq \frac{k_{i}}{2}|K_{i}=k_{i}\right)P\left(K_{i}=k_{i}\right)+\left(\frac{N+m}{n}\right){\left(\frac{\lambda }{\lambda +\lambda_{e}}\right)}^{2}. \end{array}$$

As $P(R_{i}\geq \frac{k_{i}}{2}|K_{i}\;=\;k_{i})\leq 1$, we have

(19) $$\begin{array}{c} |{\hat{P}}_{T,2}\left(N\right)\;-\;P_{T,app}^{low}\left(N\right)|\leq \left(1\;+\;\frac{N\;+\;m}{n}\right){\left(\frac{\lambda }{\lambda +\lambda_{e}}\right)}^{2}. \end{array}$$

Thus, when the gossip rate λ is sufficiently low compared to $\lambda_{e}$, the upper bound on (19) can be made arbitrarily small, making the approximation ${\hat{P}}_{T,2}\left(N\right)\approx P_{T,app}^{low}\left(N\right)$ tight. □

### Gossip Gain and an Adaptive Policy for Selecting Transmission Capacity

As a result of gossiping, when λ is low, the nodes that have the correct information $x_{s}$ as prior information keep their information as $x_{s}$ with probability $P_{T,app}^{low}\left(N\right)+P\left(K_{i}=0\right)$, which is given by ${\hat{P}}_{T,1}\left(N\right)\approx \frac{\lambda }{\lambda +\lambda_{e}}\frac{N+m}{n}+\frac{\lambda_{e}}{\lambda +\lambda_{e}}$. Thus, when λ is small, the probability ${\hat{P}}_{T,1}\left(N\right)$ can be equivalently approximated by

(20) $$\begin{array}{c} {\hat{P}}_{T,1}\left(N\right)\approx 1-\frac{n-N-m}{n}\frac{\lambda }{\lambda +\lambda_{e}}. \end{array}$$

Therefore, when the gossip rate is low, we have

$$\begin{array}{cc} E[N_{1}^{\prime }|N] & \;=\;N{\hat{P}}_{T,1}\left(N\right)=N-\frac{\lambda }{\lambda +\lambda_{e}}\frac{N}{n}\left(n\;-\;N\;-\;m\right),\\ E[N_{2}^{\prime }|N] & \;=\;\left(n\;-\;N\;\;-\;m\right){\hat{P}}_{T,2}\left(N\right)\;=\;\frac{\lambda }{\lambda \;+\;\lambda_{e}}\frac{m\;+\;N}{n}\left(n\;-\;N\;\;-\;m\right). \end{array}$$

Thus, at the end of the gossiping period, there are $E\left[N_{1}^{\prime }+N_{2}^{\prime }|N\right]+m=N+m+\frac{\lambda }{\lambda +\lambda_{e}}\frac{m}{n}\left(n-N-m\right)$ nodes that have the same information as the source $x_{s}$. If we consider the system with no gossiping, where only the source can send updates to *m* nodes, at the end of an update cycle most N+m nodes have the same information as the source. Thus, compared to the system with no gossiping, the gain (error reduction) obtained as a result of gossiping can be computed as

(21) $$\begin{array}{c} G\left(N\right)=\frac{m}{n^{2}}\left(n-N-m\right)\left(\frac{\lambda }{\lambda +\lambda_{e}}\right){\left(\frac{\lambda_{s}}{\lambda_{s}+\lambda_{e}}\right)}^{m}, \end{array}$$

which is obtained by subtracting N+m from $E[N_{1}^{\prime }+N_{2}^{\prime }|N]+m$ and dividing the result by *n* due to the definition of Δ. Note that the last term ${\left(\frac{\lambda_{s}}{\lambda_{s}+\lambda_{e}}\right)}^{m}$ in (21) is equal to the probability of entering the gossiping phase.

Let us denote the average error for a system with no gossiping (that is, λ=0) by $\Delta_{ng}$. If the gossip rate is low, the overall gain obtained from gossiping, $|\Delta -\Delta_{ng}|$, can be approximated by

(22) $$\begin{array}{c} |\Delta -\Delta_{ng}|\approx B\left(p\right)\sum_{N=0}^{n-m}\left(\pi_{0N}+\pi_{1N}\right)G\left(N\right), \end{array}$$

where B(p) is a scaling function, in terms of *p*, to represent the effect of gossiping on the steady-state distribution π.

When the gossip rate among the nodes is low, the gossip gain G(N) in (21) depends on the selection of *m*. Therefore, if the source is allowed to dynamically choose its transmission capacity *m* in terms of *N*, a natural choice is to adaptively select an *m* which maximizes the gossiping gain by solving $\frac{\partial G(N)}{\partial m}=0$. Solving this equation in terms of *m* gives us

(23) $$\begin{array}{c} m^{*}\left(N\right)=\frac{n-N}{2}-\frac{1}{\log\rho_{s}}-\sqrt{{\left(\frac{n-N}{2}\right)}^{2}+{\left(\frac{1}{\log\rho_{s}}\right)}^{2}}, \end{array}$$

where $\rho_{s}=\frac{\lambda_{s}}{\lambda_{s}+\lambda_{e}}$. (We note that $\frac{\partial G(N)}{\partial m}=0$ has two solutions. The other solution is equal to $m^{*}\left(N\right)$ in (23) except that the square-root term has a positive sign. One can show that this root is always larger than n−N and, thus, cannot be a feasible selection for *m*). In fact, it is easy to see from (23) that the optimal solution $m^{*}\left(N\right)$ always lies in the range $0\leq m^{*}\left(N\right)\leq \frac{n-N}{2}$.

When the source has infinite transmission capacity, we have $\lim_{\lambda_{s}\to \infty }m^{*}\left(N\right)=\frac{n-N}{2}$, which suggests that the source should send its information to at most half of the nodes that carry incorrect information. In the other extreme case, when the source’s transmission capacity is equal to 0, we have $\lim_{\lambda_{s}\to 0}m^{*}\left(N\right)=0$, in which case the source should not send its information to any other nodes. In general, for a given $\lambda_{s}$, $m^{*}\left(N\right)$ in (23) is a decreasing function of *N*, which means that when *N* is small, i.e., when most of the nodes have incorrect information, the source should send updates to a higher number of nodes. As *N* increases, the source should send updates to a smaller number of nodes, as most nodes carry the same information as the source. In the following section, in order to reduce the average error, we implement clustering in the gossip network.

## 5. Average Error in Clustered Networks

**Figure 2:**
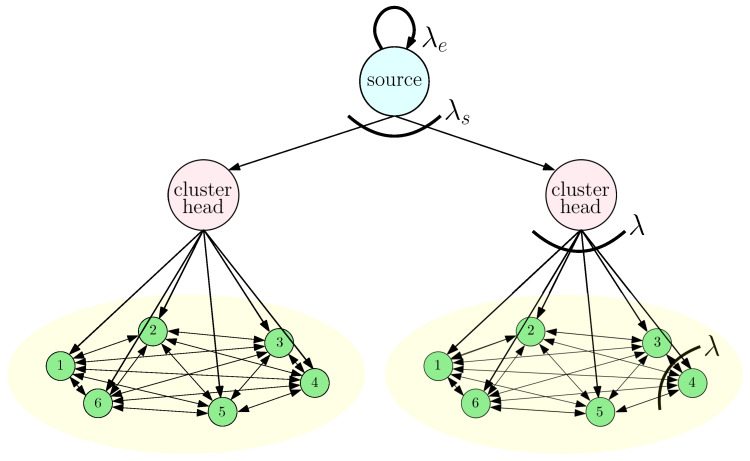
A clustered gossip network that consists of a source and $m_{s}=2$ cluster heads and fully connected $n_{c}=6$ nodes.

In this section, we explore the idea of clustering in the gossip network, in order to further reduce the average error. As illustrated in Figure 2, the gossip network is partitioned into $m_{s}$ clusters with equal cluster size $n_{c}$, i.e., without loss of generality, we assume that *n* is divisible by $m_{s}$ and, thus, we have $n_{c}=\frac{n}{m_{s}}$. Each cluster has a designated cluster head that is connected to the source directly. In the clustered network, when the information at the source is updated, instead of sending updates to individual nodes directly, the source only sends its information to cluster heads that carry different information compared to the source. Thus, the source can send its information to a smaller number of cluster heads with higher update rates. Furthermore, as the cluster heads behave like an information source of each cluster, by using cluster heads, we can increase the total update rates going from the source to the individual nodes while decreasing the total number of connections at the gossip network. The downside of clustering is that if the information is updated during transmission from the source to the cluster heads, information may not be disseminated to individual nodes in the gossip network. Thus, we need to choose the number of cluster heads $m_{s}$ optimally, to minimize the average error.

At the update cycle I(j), let the number of cluster heads that have the same information as the source be $N_{s}\left(j\right)$, where $0\leq N_{s}\left(j\right)\leq m_{s}$. Before the information at the source is updated again, the source sends updates to $K_{sc}\left(j\right)$ cluster heads with the following probability distribution:

(24) $$\begin{array}{c} P\left(K_{sc}\;=\;k_{sc}|0\;\leq \;N_{s}\;\leq \;m_{s}\right)\;=\;\left\{\begin{array}{cc} {\left(\frac{\lambda_{s}}{\lambda_{s}+\lambda_{e}}\right)}^{k_{sc}}\frac{\lambda_{e}}{\lambda_{s}+\lambda_{e}}, & \mathrm{if}\;k_{sc}\;=\;0,\cdots ,m_{s}\;-\;N_{s}\;-\;1,\\ {\left(\frac{\lambda_{s}}{\lambda_{s}+\lambda_{e}}\right)}^{m_{s}-N_{s}}, & \mathrm{if}\;k_{sc}=m_{s}-N_{s}. \end{array}\right. \end{array}$$

If $K_{sc}<m_{s}-N_{s}$, it means that the information at the source is updated before all the cluster heads obtain the information at the source. In this case, a new update cycle begins and the source starts sending information to the cluster heads again. If all the cluster heads obtain the most current information at the source, then the cluster heads start sending their information to $m_{c}$ nodes within their corresponding cluster that carries different information compared to the source with a total update rate of λ. (In this section, we introduce the cluster heads as special nodes of the network, as in [10]. However, using these special nodes may not always be possible, as they may result in additional costs for the system. Considering these factors, we take the total update rate of the cluster heads as λ, which is the same as that of the regular nodes at the clusters, which means that in the absence of these special nodes, some of the nodes in the gossip network can be used as cluster heads. As all the clusters in the network are identical, we focus on a typical cluster and obtain the average error of a node within that cluster). In a typical update cycle *I*, we define $N_{c}$ as the number of nodes that carry the same information at the source. When $N_{c}\;<\;n_{c}\;-\;m_{c}$, a cluster head sends updates to $K_{c}$ nodes with the following pmf:

(25) $$\begin{array}{c} P\left(K_{c}=k_{c}|N_{c}<n_{c}-m_{c}\right)\;=\;\left\{\begin{array}{cc} {\left(\frac{\lambda }{\lambda +\lambda_{e}}\right)}^{k_{c}}\frac{\lambda_{e}}{\lambda +\lambda_{e}}, & \mathrm{if}\;k_{c}\;=\;0,\cdots ,m_{c}\;-\;1,\\ {\left(\frac{\lambda }{\lambda +\lambda_{e}}\right)}^{m_{c}}, & \mathrm{if}\;k_{c}=m_{c}. \end{array}\right. \end{array}$$

If $N_{c}\geq n_{c}-m_{c}$, then we have

(26) $$\begin{array}{c} P\left(K_{c}=k_{c}|N_{c}\geq n_{c}-m_{c}\right)=\left\{\begin{array}{cc} {\left(\frac{\lambda }{\lambda +\lambda_{e}}\right)}^{k_{c}}\frac{\lambda_{e}}{\lambda +\lambda_{e}}, & \mathrm{if}\;k_{c}\;=\;0,\cdots ,n_{c}-N_{c}-1,\\ {\left(\frac{\lambda }{\lambda +\lambda_{e}}\right)}^{n_{c}-N_{c}}, & \mathrm{if}\;k_{c}=n_{c}-N_{c}. \end{array}\right. \end{array}$$

When $N_{c}<n_{c}-m_{c}$, if the cluster head sends updates to $K_{c}=m_{c}$ nodes, then the gossiping phase starts and all the nodes within the same cluster share their local information with one another. When the information at the source is updated, the gossiping phase ends, and the nodes that do not get information directly from the cluster head update their information based on the majority of the updates that they received during the gossiping phase.

In the next lemma, for a given state $N_{s}$, we provide the expression for the long-term average error at the nodes within the clusters.

**Lemma 1.**  *Under the proposed clustered network structure, for a given $N_{s}$, the long-term average error at the nodes within the clusters, denoted by $\Delta_{c|N_{s}}$, is given by*

(27) $$\begin{array}{c} \Delta_{c|N_{s}}=\sum_{j=0}^{n_{c}}\sum_{n^{\prime \prime }=0}^{n_{c}}\left(\pi_{0,j|N_{s}}+\pi_{1,j|N_{s}}\right)P\left(N_{c}^{\prime \prime }=n_{c}^{\prime \prime }|N_{c}=j,N_{s}\right)\frac{n_{c}-n_{c}^{\prime \prime }}{n_{c}}, \end{array}$$

*where $P(N_{c}^{\prime \prime }=n_{c}^{\prime \prime }|N_{c}=j,N_{s})$ is later provided in (30) and $\pi_{N_{s}}=[\pi_{0,0|N_{s}},\cdots ,\pi_{0,n_{c}|N_{s}},\pi_{1,0|N_{s}},$$\cdots ,\pi_{1,n_{c}|N_{s}}]$ is the row vector of the steady-state distribution of the Markov chain formed over the state space $\left(x_{s},N_{c}\right)$. The unique stationary distribution is given by the solution of $\pi_{N_{s}}=\pi_{N_{s}}P_{N_{s}}$ for a stochastic matrix $P_{N_{s}}\;\in \;R^{2\left(n_{c}\;+1\right)\times 2\left(n_{c}\;+1\right)}\;$, where the transition probabilities of $P_{N_{s}}$ can be derived by replacing $N^{\prime \prime }$, N, and n in (14) by $N_{c}^{\prime \prime }$, $N_{c}$, and $n_{c}$, respectively.*

**Proof.**  For a given $N_{s}$, the average error analysis with a clustered gossip network similarly follows from Section 3. During the gossiping phase, node *i* receives $K_{i}$ updates with the pmf in (6). Similar to (7) and (8), we can rewrite $P(R_{i}=r|K_{i}=k_{i},x_{i}=x_{s})$ and $P(R_{i}=r|K_{i}=k_{i},x_{i}\neq x_{s})$ by replacing *N* and *m* with $N_{c}$ and $m_{c}$, respectively. Then, we can define $P_{T,1}\left(N_{c}\right)$ and $P_{T,2}\left(N_{c}\right)$ as in (9) and (10), respectively. Before starting the gossiping period, $N_{c}$ nodes have $x_{s}$ and $n_{c}-N_{c}-m_{c}$ nodes have $1-x_{s}$ as their prior information. At the end of the gossiping period, we have $N_{c}^{\prime }=N_{c,1}^{\prime }+N_{c,2}^{\prime }$ nodes that have the information $x_{s}$, where $N_{c,1}^{\prime }∼\mathrm{Bin}\left(N_{c},P_{T,1}\left(N_{c}\right)\right)$ and $N_{c,2}^{\prime }∼\mathrm{Bin}\left(n_{c}-N_{c}-m_{c},P_{T,2}\left(N_{c}\right)\right)$. The pmf of $N_{c}^{\prime }$ follows from (12), with $\ell_{lower}=\max\left\{0,n_{c}^{\prime }+N_{c}+m_{c}-n_{c}\right\}$ and $\ell_{upper}=\min\left\{N_{c},n_{c}^{\prime }\right\}$.

Next, we define $N_{c}^{\prime \prime }$ to be the number of nodes in a cluster that have the same information as the source at the end of an update cycle. For a given $K_{sc}$, $N_{s}$, and $N_{c}$, similar to (13), the probability distribution of $N_{c}^{\prime \prime }$ is given by

(28) $$\begin{array}{c} \;\;\;\;\;\;P\left(N_{c}^{\prime \prime }=n_{c}^{\prime \prime }|N_{c},N_{s},K_{sc}=m_{s}-N_{s}\right)\;\;=\;\;\left\{\begin{array}{c} P(K_{c}=k_{c}|N_{c}<n_{c}-m_{c}),\\ \;\;\;\mathrm{if}\;n_{c}^{\prime \prime }=k_{c}+N_{c}<m_{c},\;\\ P\left(K_{c}=m_{c}|N_{c}<n_{c}-m_{c}\right)P\left(N_{c}^{\prime }=n_{c}^{\prime }\right),\\ \;\;\;\mathrm{if}\;m_{c}\leq n_{c}^{\prime \prime }=m_{c}+n_{c}^{\prime }<N_{c},\\ P(K_{c}=n_{c}^{\prime \prime }-N_{c}|N_{c}<n_{c}-m_{c})\\ +P\left(K_{c}\;=\;m_{c}|N_{c}\;<\;n_{c}\;-\;m_{c}\;\right)P\left(N_{c}^{\prime }\;=\;n_{c}^{\prime \prime }\;-\;m_{c}\right),\\ \;\;\;\mathrm{if}\;m_{c}\leq N_{c}\leq n_{c}^{\prime \prime }<N_{c}+m_{c},\\ P\left(K_{c}=m_{c}|N_{c}<n_{c}-m_{c}\right)P\left(N_{c}^{\prime }\;=\;n_{c}^{\prime \prime }\;-\;m_{c}\right),\\ \;\;\;\mathrm{if}\;N_{c}+m_{c}\leq n_{c}^{\prime \prime }\leq n_{c},\\ P(K_{c}=n_{c}^{\prime \prime }-N_{c}|N_{c}\geq n_{c}-m_{c}),\\ \;\;\;\mathrm{if}\;n_{c}-m_{c}\leq N_{c}\leq n_{c}^{\prime \prime }\leq n_{c}. \end{array}\right. \end{array}$$

When $K_{sc}<m_{s}-N_{s}$, we have

(29) $$\begin{array}{c} \;\;P\left(N_{c}^{\prime \prime }=n_{c}^{\prime \prime }|N_{c},N_{s},K_{sc}\;<\;m_{s}\;-\;N_{s}\right)\;=\;\left\{\begin{array}{cc} 1, & \;\mathrm{if}\;n_{c}^{\prime \prime }\;=\;N_{c},\;\\ 0, & \;\mathrm{otherwise}. \end{array}\right.\; \end{array}$$

From (28) and (29), we obtain

(30) $$\begin{array}{cc} P(N_{c}^{\prime \prime }=n_{c}^{\prime \prime }|N_{c},N_{s})= & P\left(N_{c}^{\prime \prime }\;=\;n_{c}^{\prime \prime }|N_{c},N_{s},K_{sc}\;=\;m_{s}\;-\;N_{s}\right)P\left(K_{sc}\;=\;m_{s}\;-\;N_{s}|N_{s}\right)\\ \; & \;\;\;+P\left(N_{c}^{\prime \prime }\;=\;n_{c}^{\prime \prime }|N_{c},N_{s},K_{sc}\;<\;m_{s}\;-\;N_{s}\right)P\left(K_{sc}\;<\;m_{s}\;-\;N_{s}|N_{s}\right). \end{array}$$

Finally, for a given $N_{s}$, the states $\left(x_{s},N_{c}\right)$ form a Markov chain. Similar to the average error analysis in Section 3, we label the first $n_{c}+1$ states $\left(0,0\right),\left(0,1\right),\cdots ,\left(0,n_{c}\right)$ by $1,2,\cdots ,n_{c}+1$, and the last $n_{c}+1$ states $\left(1,0\right),\left(1,1\right),\cdots ,\left(1,n_{c}\right)$ by $n_{c}+2,n_{c}+3,\cdots ,2n_{c}+2$. The stochastic matrix $P_{N_{s}}$ consists of $P_{a,b}^{c}\left(N_{s}\right)$, which denotes the probability of moving from state *a* to state *b*, and can be derived by replacing $N^{\prime \prime }$, *N*, and *n* in (14) with $N_{c}^{\prime \prime }$, $N_{c}$, and $n_{c}$, respectively. Then, we arrive at a unique stationary distribution $\pi_{N_{s}}=\pi_{N_{s}}P_{N_{s}}$ that satisfies $\sum_{i=0}^{1}\sum_{j=0}^{n_{c}}\pi_{i,j|N_{s}}=1$ and $\pi_{i,j|N_{s}}\geq 0$∀i,j. Thus, for a given $N_{s}$, we can characterize the long-term average error among all the nodes within the same cluster by (27). □

In the following theorem, we state the long-term average error of the nodes at the clusters and at the cluster heads.

**Theorem 2.**  *Under the proposed clustered network structure, the long-term average error of the nodes at the clusters, denoted by $\Delta_{c}=E\left[\Delta_{c|N_{s}}\right]$, is given by*

(31) $$\begin{array}{c} \Delta_{c}=\sum_{N_{s}=0}^{m_{s}}\left(\pi_{0,N_{s}}^{s}+\pi_{1,N_{s}}^{s}\right)\Delta_{c|N_{s}}, \end{array}$$

*where $\pi_{s}=[\pi_{0,0}^{s},\cdots ,\pi_{0,m_{s}}^{s},$$\pi_{1,0}^{s},\cdots ,\pi_{1,m_{s}}^{s}]$ is the row vector of the steady-state distribution of the Markov chain with the state space $\left(x_{s},N_{s}\right)$. The unique stationary distribution is given by the solution of $\pi_{s}=\pi_{s}P_{s}$ for a stochastic matrix $P_{s}\;\in \;R^{2\left(m_{s}\;+1\right)\times 2\left(m_{s}\;+1\right)}\;$, where the transition probabilities of $P_{s}$ are given later in (34). Furthermore, the average error of the nodes at the cluster heads, denoted by $\Delta_{s}$, is given by*

(32) $$\begin{array}{c} \Delta_{s}=\sum_{j=0}^{m_{s}}\sum_{n_{s}^{\prime \prime }=0}^{m_{s}}\left(\pi_{0,j}^{s}+\pi_{1,j}^{s}\right)P\left(N_{s}^{\prime \prime }=n_{s}^{\prime \prime }|N_{s}=j\right)\frac{m_{s}-n_{s}^{\prime \prime }}{m_{s}}, \end{array}$$

*where $P(N_{s}^{\prime \prime }=n_{s}^{\prime \prime }|N_{s}=j)$ is provided in (33).*

**Proof.**  In order to obtain the long-term average error $\Delta_{c}=E\left[\Delta_{c|N_{s}}\right]$, we need to find the probability distribution for $N_{s}$. For that, we note that in the clustered network, the information at the source and the number of cluster heads that have the same information at the source, i.e., $\left(x_{s},N_{s}\right)$, also form a Markov chain. During the source’s update transmission to the cluster heads, by using (24) we write the probability distribution for transition to state $N_{s}^{\prime \prime }$ from state $N_{s}$, as follows:

(33) $$\begin{array}{c} P\left(N_{s}^{\prime \prime }=n_{s}^{\prime \prime }|N_{s}\right)=\left\{\begin{array}{cc} {\left(\frac{\lambda_{s}}{\lambda_{s}+\lambda_{e}}\right)}^{n_{s}^{\prime \prime }-N_{s}}\left(\frac{\lambda_{e}}{\lambda_{s}+\lambda_{e}}\right), & \mathrm{if}\;N_{s}\leq n_{s}^{\prime \prime }<m_{s},\;\\ {\left(\frac{\lambda_{s}}{\lambda_{s}+\lambda_{e}}\right)}^{m_{s}-N_{s}}, & \mathrm{if}\;n_{s}^{\prime \prime }=m_{s}. \end{array}\right. \end{array}$$

The Markov chain formed by $\left(x_{s},N_{s}\right)$ has the states $\left(0,0\right),\cdots ,\left(0,m_{s}\right),\left(1,0\right)$, $\cdots ,(1,m_{s})$ where we label these states from 1 to $2(m_{s}+1)$, correspondingly. Then, the stochastic matrix $P_{s}$, consisting of $P_{a,b}^{s}$, which denotes the probability of moving from state *a* to state *b* and is given by (34). Then, we can arrive at the unique stationary distribution $\pi_{s}=\pi_{s}P_{s}$ that satisfies $\sum_{i=0}^{1}\sum_{j=0}^{m_{s}}\pi_{i,j}^{s}=1$, and $\pi_{i,j}^{s}\geq 0$, ∀i,j. Finally, by using $\Delta_{c}=E\left[\Delta_{c|N_{s}}\right]$, we obtain the average error of a node in a cluster in (31).

Similarly, the average error at the cluster heads $\Delta_{s}$ can be obtained by using (32) with the stationary distribution $\pi_{s}$ and $P(N_{s}^{\prime \prime }=n_{s}^{\prime \prime }|N_{s})$ in (33). □

(34) $$\begin{array}{c} P_{a,b}^{s}=\left\{\begin{array}{cc} \left(1-p\right)P\left(N_{s}^{\prime \prime }=b-1|N_{s}=a-1\right), & \mathrm{if}\;1\leq a\leq b\leq m_{s}+1,\\ \frac{p}{1-p}P_{a,2m_{s}+3-b}, & \mathrm{if}\;1\leq a,b-m_{s}-1\leq m_{s}+1,\;\\ \frac{p}{1-p}P_{a,2m_{s}+3-b}, & \mathrm{if}\;1\leq a-m_{s}-1,b\leq m_{s}+1,\;\\ \left(1\;-\;p\right)P\left(N_{s}^{\prime \prime }\;=\;b\;-\;m_{s}\;-\;2|N_{s}\;=\;a\;-\;m_{s}\;-\;2\right), & \mathrm{if}\;m_{s}\;+\;2\;\leq \;a,b\;\leq \;2\left(m_{s}\;+\;1\right). \end{array}\right. \end{array}$$

In general, the clustered networks can model a system where not all the nodes have access to the source directly. In a way, cluster heads constitute a small group of nodes that have the privilege of accessing the information source directly. These nodes can be considered as paid subscribers to the source, while regular nodes can have free access to the information through these paid subscribers and gossiping. Thus, looking at the average difference between the errors at the cluster heads, $\Delta_{s}$, and at the regular nodes, $\Delta_{c}$, tells us how much a regular node can increase its quality of information through subscription. We can also imagine the clustered gossip networks in a way such that if every node is connected (subscribed) to the source directly, the information quality at the individual nodes may decrease due to the limited update capacity of the source. Instead, these nodes may choose some nodes as subscribers and share the cost of the subscription. As a result, through clustering, the nodes can decrease the cost of accessing the information while increasing the overall quality of their information.

In the next section, we provide numerical results to shed light on the effects of gossiping and clustering on information dissemination.

## 6. Numerical Results

This section has three subsections: in the first one, we discuss the numerical results of the effects of various parameters, such as transmission capacity *m*, rate of information change $\lambda_{e}$, information transmission rate at the source $\lambda_{s}$, gossip rate λ, and the number of nodes *n* on information dissemination in gossip networks; in the second one, we provide simulation results to corroborate the analytical results in Section 4; in the third one, simulations illustrate the results of Section 5—that is, the effects of clustering on information dissemination.

### 6.1. Simulations for the Effects of Various System Parameters on Information Dissemination

**Figure 3:**
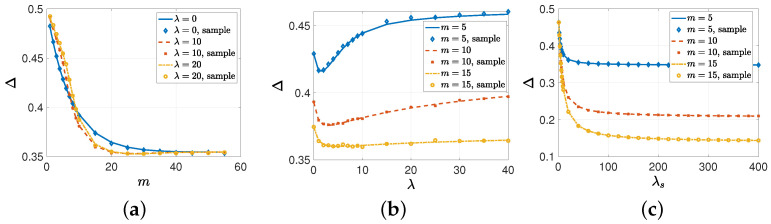
The average error Δ with respect to (**a**) *m* when λ∈{0,10,20}, (**b**) the gossip rate λ for m∈{5,10,15}, and (**c**) the source’s update rate $\lambda_{s}$ for m={5,10,15}.

**Figure 4:**
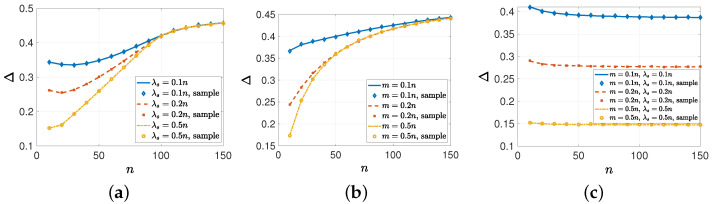
Average error Δ with respect to *n* (**a**) when $\lambda_{s}\in \left\{0.1n,0.2n,0.5n\right\}$, (**b**) when m∈{0.1n,0.2n,0.5n}, and (**c**) when m∈{0.1n,0.2n,0.5n} and $\lambda_{s}\in \left\{0.1n,0.2n,0.5n\right\}$.

In the numerical results provided in this subsection, we provide real-time simulations over 200,000 update cycles, and we provide the sample average errors with the markers in Figure 3 and Figure 4. In the first numerical study, we took p=0.4, $\lambda_{e}=1$, $\lambda_{s}=10$, and n=60. We found the average error Δ with respect to *m* when λ={0,10,20}. Note that λ=0 corresponded to the case of no gossiping among the nodes. We see in Figure 3a that when *m* was small, i.e., when the source could send updates to a small number of nodes, the average error Δ increased with gossip rate λ. As *m* was small and the information change rate p=0.4 was high, incorrect information disseminated, due to gossiping in the network. As a result, the system with no gossiping (λ=0) achieved the lowest average error. When we increased *m* sufficiently, the nodes started to have access to the same information as the source, and gossiping helped to disseminate the correct information. That is why the systems with gossiping—i.e., λ=10, 20—achieved lower average error compared to the system with no gossiping. The lowest average error Δ was achieved when m=25 for λ=10, 20 and m=55 for λ=0. Here, we also note that the average error Δ was lower when λ=10 compared to λ=20, which shows that for a given *m*, there is an optimal gossip rate that achieves the lowest average error. Finally, increasing *m* further decreased the probability of entering the gossiping phase, and that is why all the curves in Figure 3a overlap when m≥40.

In the second numerical study, we considered the same variable selections as in the previous example except that we took m={5,10,15} and changed λ from 0 to 40. We see in Figure 3b that increasing the gossip rate λ initially helped to reduce the average error Δ. Then, increasing λ further increased Δ as the incorrect information among the nodes became more available. We see in Figure 3b that the minimum average error was obtained when λ=1 for m=5, λ=3 for m=10, and λ=6 or λ=7 for m=15. We note that as the source sent updates to more nodes, the optimal gossip rate increased.

In the third numerical study, we considered p=0.2, $\lambda_{e}=1$, λ=5, and n=60. We increased $\lambda_{s}$ from 1 to 400 for m={5,10,15}. We see in Figure 3c that increasing $\lambda_{s}$ initially decreased the average error Δ faster. However, as Δ depended also on the other parameters, such as *m* and the gossip rate λ, increasing $\lambda_{s}$ further did not improve the average error Δ and it converged to 0.348 for m=5, 0.21 for m=10, and 0.144 for m=15.

In the fourth numerical study, we considered the effect of the network size *n* on the information dissemination. For that, first, we took p=0.2, $\lambda_{e}=1$, λ=10, m=8, and n={10,20,⋯,150}, and we increased $\lambda_{s}=\left\{0.1n,0.2n,0.5n\right\}$ with the network size *n*. In this case, as the network size increased, the source’s transmission rate also increased. However, we kept the total number of nodes that the source could send updated to the same, i.e., m=8 for all *n*. In Figure 4a, when $\lambda_{s}=\left\{0.1n,0.2n\right\}$, we see that the average error Δ initially decreased with *n*, as $\lambda_{s}$ was initially a primary limiting factor. Increasing *n* further increased Δ as *m* became more important. That is why all these three curves overlap each other when $\lambda_{s}$ is sufficiently large. Then, we considered a scenario where we kept $\lambda_{s}=4$ and only increased m={0.1n,0.2n,0.5n}. In Figure 4b, increasing the maximum number of nodes that the source could send updates to in an update cycle alone did not reduce Δ as *n* increased. As we increased *n*, $\lambda_{s}$ became the presiding factor, and all the curves in Figure 4b overlap. Finally, we increased both the source’s transmission rate $\lambda_{s}$ and capacity *m* with *n*, i.e., $\lambda_{s}=\left\{0.1n,0.2n,0.5n\right\}$ and m={0.1n,0.2n,0.5n}. As a result, in Figure 4c, we observe that we could achieve a constant Δ by increasing $\lambda_{s}$ and *m* proportional to *n*.

### 6.2. Simulations for High and Low Gossiping Rates

**Figure 5:**
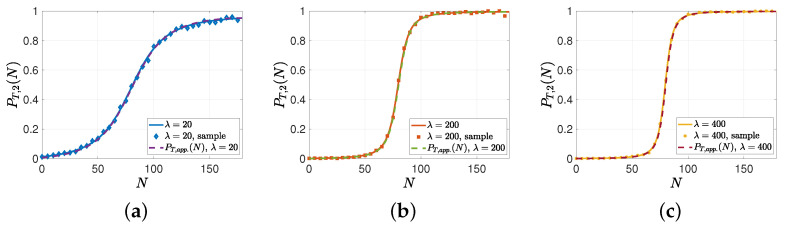
A sample evolution of $P_{T,2}\left(N\right)$, which is approximated by $P_{T,app}\left(N\right)$ in (16) when λ is high compared to $\lambda_{e}$ for (**a**) λ=20, (**b**) λ=200, and (**c**) λ=400.

In this subsection, we provide numerical results for the analysis developed for high and low gossip rates in Section 4. Here, we also ran real-time simulations over 10,000,000 update cycles. As m=20, $\lambda_{s}=2$, and $\lambda_{e}=1$, out of 10,000,000 update cycles, approximately in $10,000,000\times {\left(\frac{\lambda_{s}}{\lambda_{s}+\lambda_{e}}\right)}^{m}∼3000$ update cycles, the system entered the gossiping phase. As $P_{T,1}\left(N\right)$ and $P_{T,2}\left(N\right)$ were the probabilities of individuals that were able to obtain the source’s information as a result of gossiping, the sample averages of $P_{T,1}\left(N\right)$ and $P_{T,2}\left(N\right)$ were obtained approximately over 3000 update cycles, where the system entered the gossiping phase. In the first numerical study, we verified the analytical results in Propositions 1 and 2. For this simulation, we numerically evaluated $P_{T,2}\left(N\right)$ when n=200, m=20, $\lambda_{s}=2$, $\lambda_{e}=1$, p=0.2 for λ={20,200,400}. Then, we compared $P_{T,2}\left(N\right)$ to $P_{T,app}\left(N\right)$. In Figure 5, we observe that when λ was high compared to $\lambda_{e}$, $P_{T,2}\left(N\right)$ could be approximated well by $P_{T,app}\left(N\right)$, which was given by the summation of *Q*-functions in (16). Furthermore, due to Proposition 2, as we increased λ from 20 to 400, $P_{T,app}\left(N\right)$ and, thus, $P_{T,2}\left(N\right)$ converged to a step function, i.e., when $N\;<\;\frac{n}{2}\;-\;m\;=\;80$, we observed that $P_{T,2}\left(N\right)$ converged to 0, and when $N\;>\;\frac{n}{2}\;-\;m\;=\;80$, $P_{T,2}\left(N\right)$ converged to 1 while we had $P_{T,2}\left(80\right)=0.5$.

**Figure 6:**
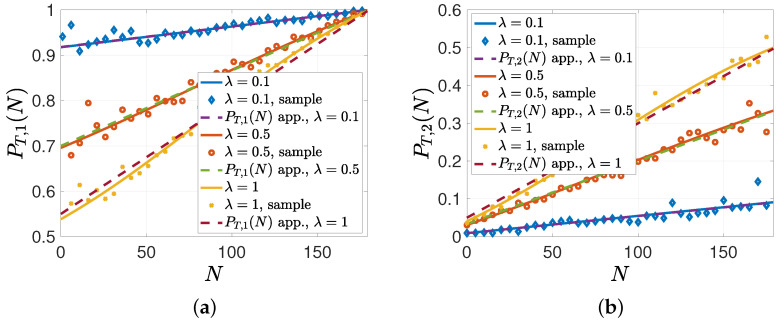
A sample evolution of (**a**) $P_{T,1}\left(N\right)$ and (**b**) $P_{T,2}\left(N\right)$ approximated by (20) and (18), respectively, when the gossiping rate is low.

In the remaining numerical studies, we considered the case when the gossip rate λ was low compared to $\lambda_{e}$. In the second simulation, we evaluated $P_{T,1}\left(N\right)$ and $P_{T,2}\left(N\right)$ with the same parameters except for λ={0.1,0.5,1}. We have shown in Proposition 3 that when λ is low compared to $\lambda_{e}$, $P_{T,2}\left(N\right)$ can be approximated by $P_{T,app}^{low}\left(N\right)$ in (18). We see in Figure 6b that when λ=0.1 and λ=0.5, $P_{T,2}\left(N\right)$ matched closely to $P_{T,app}^{low}\left(N\right)$ in (18). When $\lambda =\lambda_{e}=1$, $P_{T,2}\left(N\right)$ could still be approximated well by $P_{T,app}^{low}\left(N\right)$, but their differences started to be noticeable. Similarly, for the low gossiping rate, we see in Figure 6a that the approximation for $P_{T,1}\left(N\right)$ given in (20) was close when λ={0.1,0.5}. When the gossip rate λ was low, during the gossiping phase, the nodes either did not receive any updates, in which case they held on to their previous beliefs, or only got one update. That is why in Figure 6b, when *N* was low, $P_{T,2}\left(N\right)$, which was the probability of having the correct information as a result of gossiping for a node that had incorrect prior information, was close to 0, and then it increased with *N*.

**Figure 7:**
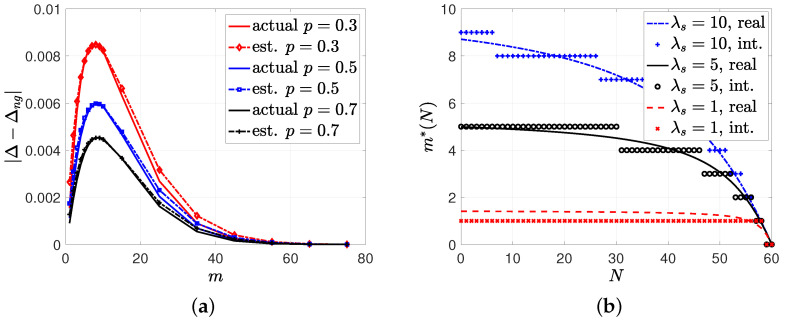
(**a**) The gossip gain $|\Delta \;-\;\Delta_{ng}|$ in (22) with regard to *m* for p={0.3,0.5,0.7}. (**b**) A sample evolution of $m^{*}\left(N\right)$ in (23) and its rounding to the nearest integer for different values of $\lambda_{s}$.

In the third simulation study, when the gossip rate was low, we numerically found the gossip gain (22), which was the difference between the average error with no gossiping $\Delta_{ng}$ and the average error with gossiping Δ. For this example, we took n=80, λ=0.4, $\lambda_{s}=10$, $\lambda_{e}=1$, and p={0.3,0.5,0.7}. We plotted $|\Delta \;-\;\Delta_{ng}|$ with respect to *m* in Figure 7a. We observed in Figure 7a that for all values of *p*, the gossip gain initially increased with *m* as the source sent correct information to a sufficient number of nodes. Then, increasing *m* further decreased the gossip gain as the probability of entering the gossiping phase decreased in an update cycle. We observe in Figure 7a that the optimum gain was obtained when m=8 for all *p* values. We note that the scaling term B(p) in (22) was equal to 1.7, 1.1, and 0.8 for p=0.2, p=0.5, and p=0.7, respectively. We also note that G(N) in (21) decreased *N* in the next update cycle with probability *p* and increased *N* with probability 1−p. Thus, the term B(p) in (22), which was the amplitude of the gossip gain, decreased with *p*.

Based on G(N) in (21), we can find the optimal *m* that maximizes the gossip gain G(N) for each *N*, which is provided as $m^{*}\left(N\right)$ in (23). So far, in this work, we have only considered the case where *m* is kept constant for all update cycles. However, $m^{*}\left(N\right)$ in (23) decreases with *N*, which suggests a policy that selects *m* adaptively, depending on *N*. In the next simulation result, we took n=60, p=0.2, λ=10, $\lambda_{e}=1$, and $\lambda_{s}=\left\{1,5,10\right\}$. In Figure 7b, we plotted $m^{*}\left(N\right)$ and their corresponding rounding to the nearest integer. We see in Figure 7b that the source sent updates to more nodes as $\lambda_{s}$ increased.

**Figure 8:**
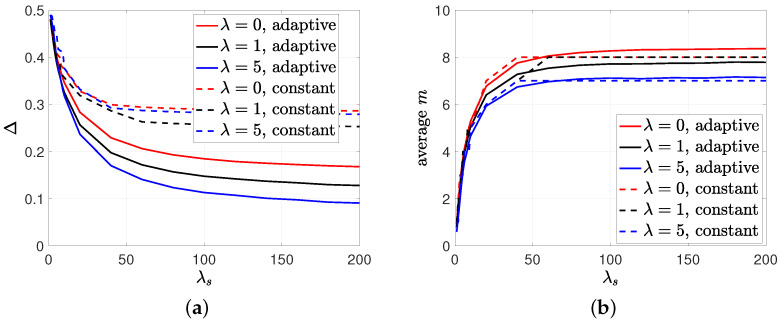
The comparison between (**a**) the average error Δ and (**b**) the average *m* for the adaptive *m* and constant *m* selection policies.

In the last simulation study, we compared the performances of the proposed adaptive policy and the constant policy for selecting *m*. We considered n=60, p=0.2, λ={0,1,5}, $\lambda_{e}=1$, and varied $\lambda_{s}$ from 1 to 200. We first implemented the adaptive-*m* transmission policy by using the nearest integer rounding of $m^{*}\left(N\right)$ in (23), which was denoted by ${\bar{m}}^{*}\left(N\right)$. We then found the stationary distribution π and calculated the average *m*, using $E\left[{\bar{m}}^{*}\right]=\sum_{j=0}^{n}\left(\pi_{0j}+\pi_{1j}\right){\bar{m}}^{*}\left(N\right)$, which is depicted in Figure 8b. In order to make a fair comparison, we took the nearest integer rounding of $E[{\bar{m}}^{*}]$, which is shown with the dashed lines in Figure 8b, and implemented the constant *m* transmission policy. We see in Figure 8a that the adaptive *m* policy (even without gossiping) achieved significantly lower average error Δ compared to the constant *m* policy. In Figure 8a, we also observe that as the gossiping took place, especially when nodes had the correct information, the average error Δ decreased with the gossip rate λ. In the adaptive *m* selection policy, we see in Figure 8b that increasing gossip rate λ not only achieved lower Δ but also decreased the source’s transmission capacity $E[{\bar{m}}^{*}]$. Even though we found this policy for low gossip rates ($\lambda <\lambda_{e}$), we observed that it was an effective transmission policy even for the higher values of λ and could achieve lower Δ compared to the constant *m* policy.

### 6.3. Simulations for the Clustered Networks

**Figure 9:**
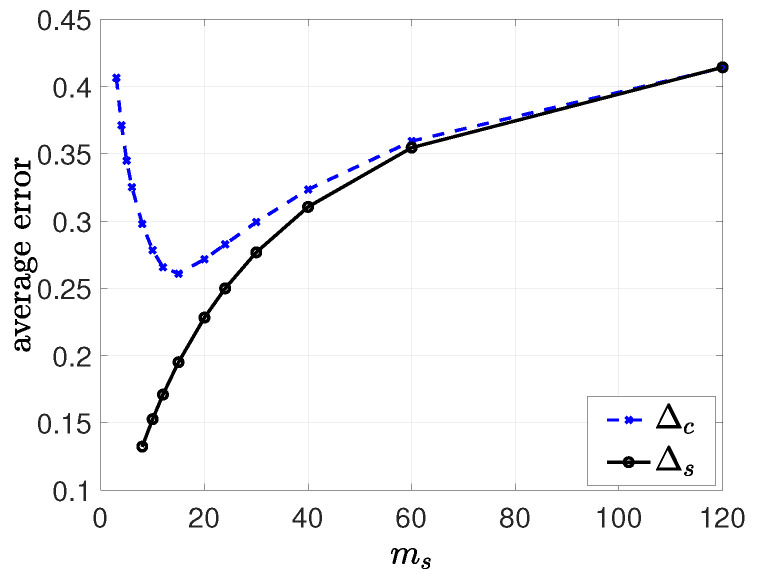
The long-term average error at the clusters, $\Delta_{c}$, and at the cluster heads, $\Delta_{s}$, as we increase the number of clusters $m_{s}$.

In this subsection, we provide the results of simulations that illustrate the effects of clustering on information dissemination. In the first numerical study, we chose λ=10, $\lambda_{s}=10$, $\lambda_{e}=1$, p=0.4, and n=120. We took $m_{c}=5$ and considered all $m_{s}$ values that could divide *n*. In Figure 9, we plotted the long-term average error at the clusters, $\Delta_{c}$, and at the cluster heads, $\Delta_{s}$. We see that increasing the number of cluster heads initially helped to reduce $\Delta_{c}$ as the update rates from the cluster heads to the nodes increased. We see in Figure 9 that the minimum $\Delta_{c}$ was achieved when $m_{s}=15$. Increasing $m_{s}$ further increased $\Delta_{c}$, as the average error at the cluster heads $\Delta_{s}$ became large.

**Figure 10:**
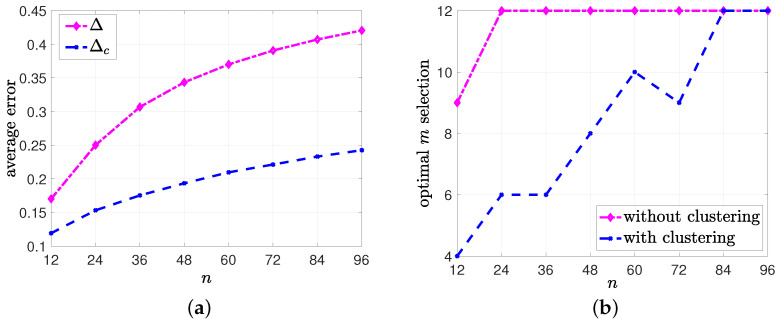
The comparison between (**a**) the average errors Δ and $\Delta_{c}$ and (**b**) the optimum *m* selections for the network models w and w/o clustering.

In the second numerical study, we compared the performances of the gossiping networks with and without clustering when the source’s transmission capacity *m* had an upper limit $m_{lim}=12$. For this numerical study, we took the same set of variables as in the first numerical study, but we increased n=12,24,⋯,96. For each *n*, we found the optimum *m* for the network model without clustering and the optimum $m_{s}$ for the clustered network that minimized the average error at the nodes. We plotted the minimum average error values in Figure 10a and the optimum *m* and $m_{s}$ selections in Figure 10b. We see in Figure 10a that the average error with clustering, $\Delta_{c}$, was smaller than the average error without clustering, Δ, for all values of *n*, although the source used its maximum capacity $m=m_{lim}$ for n≥24 in the network model without clustering, as shown in Figure 10b. For the clustered network model, the optimal number of cluster heads mostly increased with *n* and reached $m_{lim}$ for n≥84.

## 7. Conclusions and Future Directions

In this work, we considered information dissemination over gossip networks consisting of a source that keeps the most up-to-date information about a binary state of the world and *n* nodes whose common goal is to follow the binary state of the world as accurately as possible. We first characterized the equations necessary to obtain the average error Δ over all the nodes. Then, we provided analytical results for the high and low gossip rates. As information became available among the nodes in the high gossip rates, all the nodes behaved like a single node. In the low gossip case, we analyzed the gossip gain, which was the error reduction compared to the system with no gossiping, and we obtained $m^{*}\left(N\right)$, which maximized the gain. This suggests an adaptive *m* selection policy using $m^{*}\left(N\right)$, where the source sends updates to more nodes if most of them have incorrect prior information. Finally, we implemented a clustered gossiping network model and characterized the average errors at the cluster heads and at the nodes in the clusters.

We would like to note that, in this paper, information change probability *p* and update rate $\lambda_{e}$ are taken as given exogenous parameters to the source. As time passes, the source generates updates based on Poisson ticking with rate $\lambda_{e}$ among which information is reverted with rate $p\lambda_{e}$. Let us assume that $p\lambda_{e}$ is fixed (while 0<p<0.5) and *m* is constant. As $p\lambda_{e}$ is constant, information change rate over time does not change as we vary $\lambda_{e}$. When $\lambda_{e}$ is low (and, thus, *p* is relatively large), then information at the source is flipped more frequently and the update cycle duration gets longer (as a result, the probability of entering the gossiping phase is higher). As the information is flipped more often, the majority of the nodes may have incorrect information. During the gossiping phase, this may increase the average error, as incorrect information may be disseminated further in the network. On the other hand, when $\lambda_{e}$ is high while *p* is low, the information at the source is updated more frequently, but information does not get mutated much. In this case, the probability of entering the gossiping phase decreases and, thus, the system may not benefit from gossiping. Therefore, for a fixed $p\lambda_{e}$, there should be an optimal *p* and $\lambda_{e}$ selection that minimizes the average error. We leave the optimization problem over $\lambda_{e}$ as a future research direction.

As a future direction of research, one could consider the problem where the information at the source can take k>2 different values based on a known pmf. Furthermore, here we have considered only fully connected networks, and extending these results to arbitrarily connected networks could be another interesting direction. One could consider a setting where the source does not have access to the prior information on the nodes, and has to select nodes randomly. In addition to the real-time simulation results, we would like to test our results with the real-world datasets provided in [25]. Finally, one can consider a setting where, although some nodes have the most accurate information, they maliciously send incorrect information to others during the gossiping phase, thus increasing average error.

## Data Availability

No new data were created or analyzed in this study. Data sharing is not applicable to this article.
